# Aortic Counterpulsation Therapy in Patients with Advanced Heart Failure: Analysis of the TBRIDGE Registry

**DOI:** 10.5935/abc.20150147

**Published:** 2016-01

**Authors:** Cristiano Guedes Bezerra, Eduardo Leal Adam, Mariana Lins Baptista, Giuliano Serafino Ciambelli, Liliane Kopel, Claudia Bernoche, Leonardo Nicolau Geisler Daud Lopes, Milena Frota Macatrão-Costa, Breno de Alencar Araripe Falcão, Silvia Gelas Lage

**Affiliations:** Instituto do Coração do Hospital das Clínicas da Faculdade de Medicina da Universidade de São Paulo (InCor HC FMUSP), São Paulo, SP - Brazil

**Keywords:** Shock, Cardiogenic / mortality, Heart Failure, Chagas Cardiomyopathy, Intra-Aortic Balloon Pump, Heart Transplanation, Counterpulsation

## Abstract

**Background:**

The use of aortic counterpulsation therapy in advanced heart failure is controversial.

**Objectives:**

To evaluate the hemodynamic and metabolic effects of intra-aortic balloon pump (IABP) and its impact on 30-day mortality in patients with heart failure.

**Methods:**

Historical prospective, unicentric study to evaluate all patients treated with IABP betwen August/2008 and July/2013, included in an institutional registry named TBRIDGE (The Brazilian Registry of Intra-aortic balloon pump in Decompensated heart failure - Global Evaluation). We analyzed changes in oxygen central venous saturation (ScvO_2_), arterial lactate, and use of vasoactive drugs at 48 hours after IABP insertion. The 30-day mortality was estimated by the Kaplan-Meier method and diferences in subgroups were evaluated by the Log-rank test.

**Results:**

A total of 223 patients (mean age 49 ± 14 years) were included. Mean left ventricle ejection fraction was 24 ± 10%, and 30% of patients had Chagas disease. Compared with pre-IABP insertion, we observed an increase in ScvO_2_ (50.5% vs. 65.5%, p < 0.001) and use of nitroprusside (33.6% vs. 47.5%, p < 0.001), and a decrease in lactate levels (31.4 vs. 16.7 mg/dL, p < 0.001) and use of vasopressors (36.3% vs. 25.6%, p = 0.003) after IABP insertion. Thirty-day survival was 69%, with lower mortality in Chagas disease patients compared without the disease (p = 0.008).

**Conclusion:**

After 48 hours of use, IABP promoted changes in the use of vasoactive drugs, improved tissue perfusion. Chagas etiology was associated with lower 30-day mortality. Aortic counterpulsation therapy is an effective method of circulatory support for patients waiting for heart transplantation.

## Introduction

Cardiogenic shock is a clinical condition with a high mortality rate.^1,2^ Patients with severe ventricular dysfunction and advanced heart failure are frequently referred to intensive care units (ICUs) for hemodynamic support. Despite pharmacological therapy including diuretics, vasodilators and inotropic agents, many of these patients persists in shock, demanding support until recovery or heart transplantation.

Introduced in the 1960s, the intra-aortic balloon pump (IABP) remains the most widely used circulatory assist device in cardiogenic shock.^3,4^ Its effectiveness for the management of these patients is based on the positive hemodynamic effects on cardiac output, coronary perfusion and left ventricular afterload, in addition to its suitability to the intensive care setting.^5,6^

Although there are no randomized controlled trials showing a reduction in mortality, the IABP has been indicated to patients with acute coronary syndromes and hemodynamic instability until recovery of stunned myocardium.^7-9^ Its indication in advanced cardiomyopathy also lacks evidence, although is related to clinical stabilization and maintenance of tissue perfusion during advanced stages of the disease. The aims of this study were to evaluate hemodynamic and metabolic effects of IABP and 30-day mortality in patients with advanced cardiomyopathy.

## Methods

Historical prospective, unicentric study, performed to evaluate all patients treated with IABP between August/2008 and July/2013, in a cardiac ICU dedicated to heart failure patients. Data were obtained from an institutional registry named TBRIDGE (The Brazilian Registry of Intra-aortic ballon pump in Decompensated heart failure - Global Evaluation), created to evaluate the IABP performance on circulatory assistance in patients with advanced cardiomyopathy based on information collected from patients' electronic medical records. We assessed the central venous oxygen saturation (ScvO_2_), pH, bicarbonate, base excess, arterial lactate, hemoglobin, white blood cell count, platelet count, urea, creatinine, sodium, brain natriuretic peptide (BNP), C-reactive protein (CRP), and echocardiographic data - left ventricular ejection fraction (LVEF), left ventricle end-diastolic diameter, left ventricle end-systolic diameter, pulmonary artery systolic pressure, presence and degree of right ventricular dysfunction, presence and degree of mitral regurgitation. We also investigated the percentage of patients using vasoactive drugs (dobutamine, milrinone, sodium nitroprusside) and vasopressors (norepinephrine and dopamine). Patients were classified according to the level of respiratory support (room air, nasal cannula oxygen, non-invasive positive pressure ventilation, invasive mechanical ventilation) and severity of renal dysfunction. Renal failure was defined as need for hemodialysis or estimated creatinine clearance < 60 mL/min, calculated by the Cockroft-Gault formula. Clinical and laboratory data immediately before (pre-IABP) and 48 hours after the insertion of IABP (post-IABP) were compared.

We evaluated 30-day mortality, rate of heart transplantation, readmissions after hospital discharge, IABP-related complications (pseudoaneurysm, bleeding, arteriovenous fistula, arterial and venous embolism), and the rate of anticoagulant use.

Quantitative variables were expressed as mean and standard deviation and median and interquartile range (IQR), as appropriate. Qualitative variables were expressed as absolute frequencies and percentages. Pre- and post-IABP quantitative data were compared by paired Student's t-test (for normal distribution data) or by the Wilcoxon test (when assumption of normal distribution was rejected), and qualitative data were compared by the McNemar test.

Survival curve was calculated by the Kaplan-Meier method and diferences in subgroups were evaluated by the Log-rank test. Clinical assessment of patients in the late follow-up was based on the last outpatient visit data contained in their medical records. A p-value < 0.05 was considered to be statistically significant. Analysis of data was performed using the SPSS software, version 17.0 (SPSS Inc., Chicago, USA). The study was approved by the local ethics committee.

## Results

A total of 2,892 patients were admitted to the cardiac ICU, and 223 (7.7%) received 302 IABPs, during the five-year period of the study. Clinical features are described in table 1. The leading etiologies of heart failure were Chagas disease (30%), ischemic cadiomyopathy (29%) and idiopathic dilated cardiomyopathy (15%) (Table 2). The most common indication for IABP was low cardiac output syndrome (93%), followed by refractory angina (1.7%), electrical storm (2.2%), and support for high-risk procedures (3.1%).

**Table 1:** Characteristics of patients treated with intra-aortic balloon pump

**Characteristics**	
Mean age (years)	49.3 ± 14.6
Male - n (%)	162 (72.6)
Hypertension - n (%)	90 (40.3)
Diabetes mellitus - n (%)	32 (13.9)
Dyslipidemia - n (%)	78 (34.9)
Smoking - n (%)	75 (33.6)
Cerebrovascular disease - n (%)	29 (13)
Chronic obstructive pulmonary disease - n (%)	6 (2.7)
Hypothyroidism - n (%)	25 (11.2)
Peripheral vascular insufficiency - n (%)	8 (3.6)
Previous percutaneous coronary intervention - n (%)	40 (17.9)
Previous coronary artery bypass grafting - n (%)	21 (9.4)
Previous heart transplantation - n (%)	4 (1.7)
Permanent atrial fibrillation - n (%)	67 (30)
Implantable cardioverter defibrillator - n (%)	13 (5.8)
Pacemaker - n (%)	21 (9.4)
Cardiac resynchronization therapy - n (%)	19 (8.5)
Cancer - n (%)	9 (4)
Primary valvular disease - n (%)	11 (4.9)

**Table 2:** Causes of heart failure

**Causes of heart failure**	**%**
Chagas disease	30.1
Ischemic cardiomyopathy	29
Idiopathic dilated cardiomyopathy	15.3
Valvular cardiomyopathy	6.3
Viral myocarditis	3.6
Alcoholic cardiomyopathy	3.2
Cardiomyopathy after chemotherapy	3.2
Others	9.3

The median time of hemodynamic support with IABP was 10 days (IQR: 4-22.5). The longest time on IABP was 263 days. Fifty-two patients (23.3%) used two or more IABP devices during hospitalization.

The mean LVEF assessed by echocardiogram was 24 ± 10%, with left ventricular diastolic diameter of 69 mm. Moderate or severe mitral regurgitation was observed in 62% of patients, and moderate or severe right ventricular dysfunction was found in 70.8% of patients (Table 3).

**Table 3:** Echocardiographic characteristics

**Echocardiographic data**	
Left ventricle ejection fraction (%)	24.4 ± 10
Left ventricle diastolic diameter- mm	69.4 ± 12
Left ventricle systolic diameter - mm	60.8 ± 13
Pulmonary artery systolic pressure - mmHg	50.6 ± 12
Moderate to severe mitral regurgitation (% of patients)	62
Moderate to severe right ventricle dysfunction (% of patients)	70.8

Laboratory data before and 48 hours after the insertion of IABP are presented in table 4. Microhemodynamic parameters improved post-IABP as compared with pre-IABP values, such as a decrease in serum lactate (32.9 *vs.* 17.1 mg/dL, p < 0.01), and an increase in ScvO_2_ (50.6 *vs.* 66%, p < 0.01), pH (7.37 *vs*. 7.39, p < 0.001), serum bicarbonate (21.1 *vs*. 23.8 mg/dl, p < 0.001) and base excess (-3.31 *vs*. -0.5, p < 0.001). Although creatinine levels did not significantly change after the IABP, a significant decrease in the levels of urea was observed.

**Table 4:** Comparison of laboratory data between pre- and post-insertion of intra-aortic balloon pump (IABP)

**Serum parameters**	**Pre-IABP insertion**	**Post-IABP insertion**	**p value**
	**Mean ± DP**	**Mean ± DP**	
Hemoglobin (g/dL)	11 ± 2	10 ± 1.66	< 0.01
White blood cell count (/mm^3^)	10105 ± 4871	10164 ± 5350	0.92
Platelet count (/mm^3^)	211444 ± 82599	162069 ± 71889	< 0.01
Urea (mg/dL)	83 ± 46	75.6 ± 43	
Creatinine (mg/dL)	2.19 ± 1.3	2.04 ± 1.28	
pH	7.36 ± 0.09	7.39 ± 0.06	< 0.01
Bicarbonate (mmol/L)	20.9 ± 5.2	23.7 ± 4.3	< 0.01
Base excess (mmol/L)	-3.55 ± 5.54	-0.62 ± 4.43	< 0.01
ScvO_2_ (%)	50.6 ± 14.8	66 ± 12.9	< 0.01
Arterial lactate (mg/dL)	32.9 ± 28.9	17.1 ± 16.2	< 0.01
Sodium (mEq/L)	132 ± 5		
BNP (pg/mL)	2213 ± 1604		
PCR (mg/dL)	66.7 ± 63		

ScvO_2_: Central venous oxygen saturation; BNP: Brain natriuretic peptide; PCR: C-reactive protein.

After 48 hours of IABP insertion, we found an increased use of the vasodilator sodium nitroprusside (33.7 *vs* 47.5%, p = 0.0002) and reduced use of norepinephrine and dopamine (36.2 *vs* 25.6%, p = 0.0036). No significant differences in the rate of dobutamine or milrinone use were found (Table 5).

**Table 5:** Comparison of the frequency of use of vasoactive drugs by the patients between pre and post-insertion of intra-aortic balloon pump (IABP)

**Vasoactive drugs**	**Pre-IABP insertion**		**Post-IABP insertion**		**p value**
	**n**	**%**	**n**	**%**	
Norepinephrine	66	32.6	57	28.2	0.22
Norepinephrine + Dopamine	81	36.3	57	25.5	0.0036
Dobutamine	192	95	192	95	1
Sodium nitroprusside	68	33.6	96	47.5	0.0002
Milrinone	23	11.4	23	11.4	1

The degree of respiratory support did not change after the IABP as compared with pre-IABP. Fifty-one (23%) patients were on room air, 84 (38%) received high flow nasal cannula oxygen, 23 (10%) were on non-invasive positive pressure ventilation and 65 (29%) on mechanical ventilation.

Before IABP, 172 (77.1%) patients had renal failure, and 20 (8%) of them received hemodialysis. A significant decrease in the number of patients with renal failure, on or not on hemodialysis, was observed after IABP as compared with pre-IABP (77.1% *vs*. 62.33%, p = 0.001).

**Figure 1:**
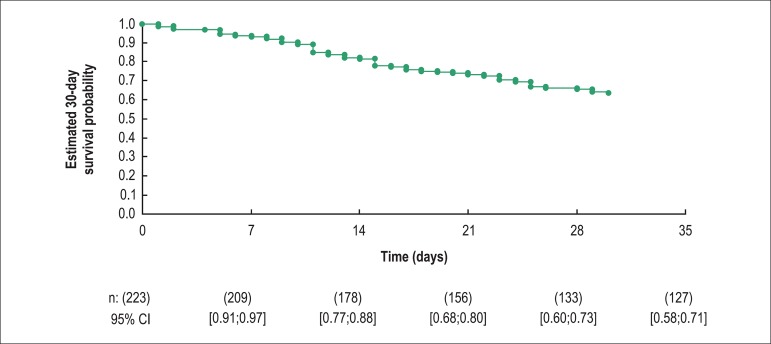
Thirty-day survival curve.

**Figure 2:**
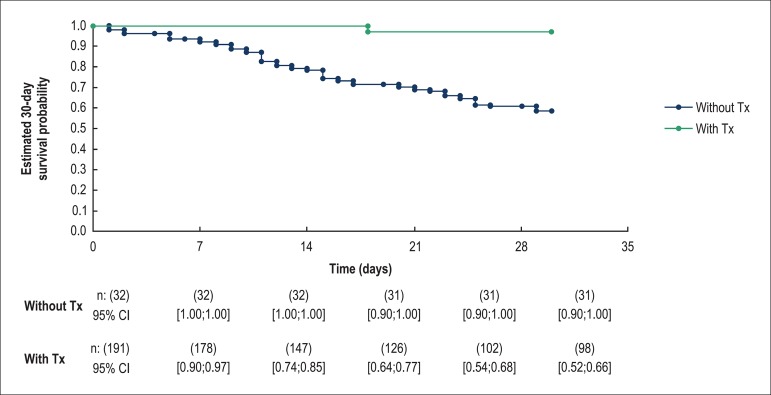
Comparison of 30-day mortality between transplant patients (with Tx) and non-transplant patients (without Tx). n: number of patients at risk; 95%CI: 95% confidence interval. P-value was calculated using the Log-rank test: p < 0.001.

**Figure 3:**
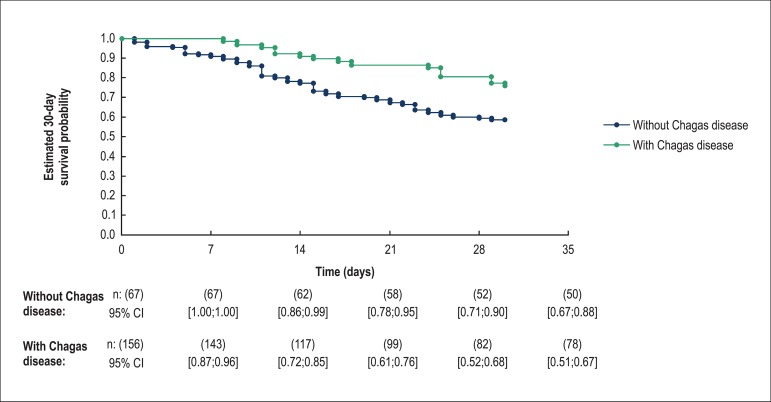
Comparison of 30-day mortality between patients with Chagas disease and other cardiomyopathies. n: number of patients at risk; 95% CI: 95% confidence interval. p value was calculated using the Log-rank test: p = 0.008.

The median duration of hospitalization was 37 days. Thirty-day survival was 63.9% (Figure 1). Heart transplantation was performed in 14% of patients. In-hospital mortality was 75%, 82% for those who did not receive heart transplantation and 29% for those who underwent transplantation (Figure 2). The median time from the IABP insertion to the transplantation was 28 days, with maximum time of 217 days. A readmission rate of 80% and late mortality rate of 20% was found in the follow-up of 56 discharged patients. Patients with Chagas heart disease (Figure 3) had a greater 30-day survival than patients with other causes (77.5% *vs.* 58.7%, p = 0.008).

With respect to the safety of IABP, a platelet count fall greater than 50,000/mm^3^ occurred in 39% of patients, and absolute thrombocytopenia in only 15% of them. A decrease in hemoglobin > 3 g/dL was observed in 8% of patients, and only one patient was diagnosed with retroperitoneal hematoma. Arterial and venous thromboembolic events occurred in 6% of patients, including stroke, peripheral arterial and venous thromboembolism.

Most patients (50.6%) did not receive full anticoagulation; 2,7% of patients received anticoagulation due to the presence of the IABP and 46.6% for other reasons (venous thromboembolism, atrial fibrillation, stroke, intracavitary thrombus).

## Discussion

This study evaluated clinical data of patients with advanced cardiomyopathy admitted to a cardiac ICU. Differently from previous studies on cardiogenic shock, wich focus on acute myocardial infarction,^10,11^ our population was composed predominantly of patients with advanced chronic heart failure, refractory to pharmacological therapies. The advanced stage of cardiomyopathy in our study population was characterized by severe left ventricular dysfunction, which was frequently associated with right ventricular dysfunction, hyponatremia, pulmonary arterial hypertension, and increased BNP levels.

The use of IABP reduced the use of vasopressors, which are known to increase the afterload in left ventricular dysfunction. There was also a significant increase in the use of sodium nitroprusside, a potent arterial vasodilator, beneficial to heart failure patients. Laboratorial data markedly improved after the IABP insertion, with reduction of lactate and urea levels, and increase of ScvO2, pH, bicarbonate and base excess, which may be explained by the effects of IABP on the hemodynamic profile and on the use of vasoactive drugs.

There was a high prevalence of renal failure in our study population. Although the mean creatinine levels did not change, both the percentage of patients with renal failure and urea serum concentrations significantly decreased after 48 hours of aortic counterpulsation. Further studies are needed to evaluate the role of IABP on the maintenance and recovery of renal function in patients with cardiogenic shock.

Heart failure caused by Chagas disease has been associated with unfavorable prognosis.^12,13^ In our study, however, chances of early survival were higher in Chagas disease patients using the IABP compared with patients with other etiologies of heart failure. Data from the literature show that transplant patients with Chagas disease had better prognosis than those without the disease, with survival rates of 83%, 71%, 57% and 46% at 1 month, 1 year, 4 years and 10 years of follow-up, respectively.^14^ The paradox of greater survival of Chagas disease patients after heart transplantation was also demonstrated in a Brazilian muti-centric study involving 720 patients.^15^

Despite an initial clinical improvement following IABP insertion, more than 80% of non-transplant patients died during hospitalization, which emphasizes the need for therapies to improve prognosis. This was highlighted by the difference in mortality between transplant and non-transplant patients.

Another important consideration was the number of patients (50.6%) not receiving therapeutic anticoagulation and the low incidence of thromboembolic events. The use of IABP in this population reinforces its role as prolonged circulatory support in patients with advanced cardiomyopathy, candidates for transplantation. One patient used the IABP for 217 days until receiving a successful transplant. In agreement with the largest randomized study on the subject, the IABP-SHOCK II trial which involved 600 patients, the use of IABP was considered safe, as it did not increase the risk of complications, such as peripheral ischemia, infection or bleeding.^11^

Finally, this is the largest registry of circulatory support in advanced cardiomyopathy patients in Brazil. Nevertheless, due to its observational and unicentric design, this study aimed to generate hypotheses that may contribute to the management of these high mortality risk patients.

## Conclusion

IABP showed beneficial effects in the first 48 hours, promoting changes in the vasoactive drugs regimen and improving tissue perfusion. Chagas etiology was associated with lower 30-day mortality. Aortic counterpulsation therapy is an effective alternative method of circulatory support for patients waiting for heart transplantation.
